# Idiopathic Hypogonadotropic Hypogonadism Caused by Inactivating Mutations in SRA1

**DOI:** 10.4274/jcrpe.3248

**Published:** 2016-06-06

**Authors:** Leman Damla Kotan, Charlton Cooper, Şükran Darcan, Ian M. Carr, Samim Özen, Yi Yan, Mohammad K. Hamedani, Fatih Gürbüz, Eda Mengen, İhsan Turan, Ayça Ulubay, Gamze Akkuş, Bilgin Yüksel, A. Kemal Topaloğlu, Etienne Leygue

**Affiliations:** 1 Çukurova University Faculty of Medicine, Department of Pediatrics, Division of Pediatric Endocrinology, Adana, Turkey; 2 University of Manitoba, Manitoba Institute of Cell Biology, Winnipeg, Manitoba, Canada; 3 Ege University Faculty of Medicine, Department of Pediatrics, Division of Pediatric Endocrinology, İzmir, Turkey; 4 University of Leeds, Institute of Biomedical and Clinical Sciences, Section of Genetics, Leeds, United Kingdom; 5 Çukurova University Faculty of Medicine, Department of Forensic Medicine, Adana, Turkey; 6 Çukurova University Faculty of Medicine, Division of Endocrinology and Metabolism, Adana, Turkey

**Keywords:** Hypogonadotropic hypogonadism, puberty, SRA1, PNPLA6, mutation

## Abstract

**Objective:** What initiates the pubertal process in humans and other mammals is still unknown. We hypothesized that gene(s) taking roles in triggering human puberty may be identified by studying a cohort of idiopathic hypogonadotropic hypogonadism (IHH).

**Methods:** A cohort of IHH cases was studied based on autozygosity mapping coupled with whole exome sequencing.

**Results:** Our studies revealed three independent families in which IHH/delayed puberty is associated with inactivating SRA1 variants. SRA1 was the first gene to be identified to function through its protein as well as noncoding functional ribonucleic acid products. These products act as co-regulators of nuclear receptors including sex steroid receptors as well as SF-1 and LRH-1, the master regulators of steroidogenesis. Functional studies with a mutant SRA1 construct showed a reduced co-activation of ligand-dependent activity of the estrogen receptor alpha, as assessed by luciferase reporter assay in HeLa cells.

**Conclusion:** Our findings strongly suggest that SRA1 gene function is required for initiation of puberty in humans. Furthermore, SRA1 with its alternative products and functionality may provide a potential explanation for the versatility and complexity of the pubertal process.

## WHAT IS ALREADY KNOWN ON THIS TOPIC?

Pubertal development is thought to be a result of a complex interplay among genes and environmental factors including nutrition.

## WHAT THIS STUDY ADDS?

SRA1 gene function is required for the initiation of puberty in humans.

## INTRODUCTION

What initiates the pubertal process in humans and other mammals is still an enigmatic question (1). The hallmark of puberty is reemergence of a pulsatile gonadotropin-releasing hormone (GnRH) release from the hypothalamus driving the pituitary gonadotropes to secrete luteinizing hormone (LH) and follicle-stimulating hormone (FSH), which act in concert to stimulate the gonads to bring about sex hormone secretion and gametogenesis. Normosmic idiopathic hypogonadotropic hypogonadism (IHH) is characterized by failure to develop secondary sexual characteristics and a mature reproductive system due to defects in the central part of the hypothalamo-pituitary-gonadal axis. In an effort to gain a greater understanding into the elusive pubertal process, our laboratory has undertaken a search for genes playing important roles in the generation of gonadotropin secretion in a cohort of familial IHH cases via autozygosity mapping. Our previous seminal descriptions of the mutated genes, TAC3, TACR3 (2), and KISS1 (3) in this same patient cohort led the way to the characterization of the GnRH pulse generator (4). Along the same line, we hypothesize that gene(s), whose products trigger the GnRH pulse generator to restart ticking at the usual time of puberty, can also be identified via autozygosity mapping together with whole exome sequencing.

## METHODS

As an overall genetic sequencing strategy, we screen probands from consanguineous multiplex pedigrees for known IHH genes in our IHH cohort. If no mutation is found, then we perform autozygosity mapping based on either single nucleotide polymorphism (SNP) microarray genotyping or (lately) whole exome sequencing. Once we identify a likely candidate gene, we then screen the probands for that gene in the entire cohort either with Sanger sequencing or whole exome sequencing.

## CASE REPORTS

**Family 1**

The proband (II-2), a 25-year-old male, grew and developed normally until his early-to-mid-teen years. At the age of 15, he presented with a chief complaint of absent pubertal development. His past medical history was remarkable for undescended testicles, for which he received human chorionic gonadotropin treatment. His testicular volumes were 3 mL bilaterally. His basal and stimulated gonadotropin levels as well as testosterone concentrations remained prepubertal.

The affected sibling (II-1) is a 30-year-old female. She grew and developed normally until her early-to-mid-teen years. At the age of 15, she presented with absent breast development and lack of menses. She had Tanner stage 1 breast development and stage 2 axillary and pubic hair. Her bone age then was 13 years. A pelvic ultrasonography showed a hypoplastic uterus and ovaries.

The parents are healthy and paternal cousins of Turkish origin. The mother experienced menarche at age 12 years, and the father started shaving at age 14 years.

**Family 2**

The proband (II-3), now an 18-year-old man, first presented to us at age 13 years with a small penis for which he had been given some human menopausal gonadotropin injections elsewhere. His past medical history was remarkable for undescended testicles and inguinal hernia. At age 14, his testicular volumes were 3 mL bilaterally, with stretched penile length of 4 cm. While having a bone age of 13, his gonadotropin and testosterone levels were prepubertal. Shortly afterwards, his testicular volumes increased to pubertal levels with corresponding penile growth and pubertal hormone levels. Both of his sisters have gone through puberty in time.

The parents are healthy, unrelated, and of Turkish origin. The mother experienced menarche at age 12 years, and the father started shaving at age 14 years.

**Family 3**

The proband (II-3) is a 21-year-old male, who grew and developed normally until his early-to-mid-teen years. At the age of 16, he presented with absent pubertal development. His past medical history was unremarkable. His testicular volumes were 2 mL bilaterally. His bone age was 12 years at presentation. His basal and stimulated gonadotropin levels as well as testosterone concentrations remained prepubertal.

The affected sibling (II-4) is a 17-year-old male. He grew and developed normally until his early-to-mid-teen years. At the age of 12.5 years, he presented with absent pubertal development. His past medical history was remarkable for a small penis and undescended testicles for which he received human chorionic gonadotropin treatment and subsequent orchiopexia at age 4. His right testicle was reportedly not found at the intervention. His bone age was 11.5 years at presentation to us. Later at age 14 years, he has spontaneously started pubertal development to become a young adult with normal hormonal values.

The parents are healthy, unrelated, and of Kurdish origin. The mother experienced menarche at age 12.5 years, and the father started shaving at age 13.5 years.

The clinical and hormonal features of the affected individuals in the three families are shown in Table 1.

**Figure 1:**
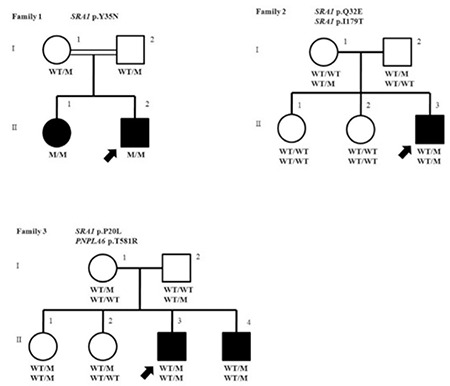
Pedigree profile and corresponding SRA1 and PNPLA6 mutations in the three families. Pedigrees are shown to indicate phenotypes and genotypes among family members. Filled circles indicate affected girls or women, open circles unaffected female family members, filled squares affected male family members, and open squares unaffected male family members. The double line indicates consanguinity. Under each available individual is the SRA1 and PNPLA6 gene genotype with M indicating mutant and WT indicating wild type

The pedigrees of the families are shown along with their genotypes in Figure 1. The patients are otherwise healthy and have a normal sense of smell. They have otherwise normal anterior pituitary functions. In consideration of the known roles of the SRA1 with other nuclear receptors, any disorders associated with a potential dysfunction of these receptors were actively ruled out. Thus, thyroid receptor was ruled out by normal thyroid function test, PPAR g by normal fasting blood glucose and Hemoglobin A1c, corticosteroid receptors by normal 8 AM cortisol and adrenocorticotropic hormone (ACTH) levels, retinoid receptors by direct examination of the retina by an ophthalmologist, and dilated cardiomyopathy due to abnormal myogenesis by echocardiography and a cardiological examination.

The Ethics Committee of the Çukurova University Faculty of Medicine approved this study, and informed consent was obtained from each participant or from the parents.

**Hormonal measurements in the affected individuals:** Plasma ACTH, serum LH, FSH, estradiol, dehydroepiandrosterone sulfate, cortisol, and testosterone levels were analyzed by commercial kits based on solid-phase, two-site sequential, or competitive chemiluminescent immunometric assay (Beckman Coulter).

A GnRH stimulation test was performed on the proband of the Family 1 by injecting 0.1 mg GnRH intravenously and drawing blood samples at 0, 15, 30, 45, 60, and 75 minutes for FSH and LH determinations.

A prolonged GnRH stimulation test was also performed on the proband of the Family 1. A daily subcutaneous injection of GnRH at 0.1 mg for one week was administered and on the 7th day, plasma FSH and LH levels were determined 30, 45, and 60 minutes after the injection.

**Screening of known genes by Sanger sequencing:** Known or strong candidate genes for IHH and Kallmann syndrome including GNRHR, GNRH1, LHB, FSHB, KISS1R, KISS1, TAC3, TACR3, KAL1, PROK2, PROK2R, and FGFR1 were screened by automated Sanger sequencing (5). Briefly, polymerase chain reaction-amplified exons and splice junctions were sequenced on an ABI PRISM 3130 autosequencer (Applied Biosystems).

**Genome-wide single nucleotide polymorphism analysis:** For genome-wide SNP analysis, we used 250 K NspI SNP microarrays (Affymetrix) and analyzed data using AutoSNPa software (AutoSNPa.org) to identify autozygous regions in Family 1.

**Whole exome sequencing:** Samples were prepared as an Illumina sequencing library, and in the second step, the sequencing libraries were enriched for the desired target using the Illumina Exome Enrichment protocol. The captured libraries were sequenced using Illumina HiSeq 2000 Sequencer (Macrogen, Seoul, Korea). The reads are mapped against UCSC hg19.

**Site-directed mutagenesis:** SRA-D7 and SRA-WT constructs were previously described (6). SRA-Y35N was generated using synthetic oligonucleotides and the QuickChangeII site directed mutagenesis kit (Agilent Technologies) using SRA-WT vector as template following manufacturer’s protocol.

**Western Blot and immunofluorescent microscopy:** Western Blot and Immunofluorescent Microscopy were performed as previously described (7).

**Luciferase assay:** Twenty-four hours prior to transfection, HeLa cells were seeded into 24 well dishes (7.5x105 cells/well) containing 5% CS-FBS phenol-red free DMEM containing 4.5 g/L D-glucose and 2 mM L-glutamine. Cells were then co-transfected with constructs expressing PS2-ERE (0.4 ug), ERa (0.04 ug), Renilla (0.02 ug), and various SRA (0.34 ug) using Lipofectamine 2000 (Life Technologies) according to the manufacturer’s protocol. The next day, the medium was changed and replaced with 5% CS-FBS DMEM containing either ethanol vehicle (cont-E) or 10 nM beta-estradiol [+E (Sigma)] for an additional 24 hours before lysis in 100 uL 1xPLB buffer (Promega). Renilla luciferase and luciferase activities were measured using SpectraMaxL Luminometer (Molecular Devices) and SoftMax Pro software using Dual Luciferase Reporter Assay System (Promega) reagents according to the manufacturer’s instructions. For each constructs, readings were normalized to control. Results represent the average of four independent experiments performed in triplicate.

**Statistical Analysis**

HeLa cell transfected with SRA-WT and mutant constructs were compared to control cells transfected with SRA-D7 construct in the absence or presence of estradiol using unpaired 2-tail student’s t-test. Graphpad prism 5 software was used for all statistical analyses.

## RESULTS

Hormonal results as shown in Table 1 are consistent with the diagnosis of IHH.

**Figure 2:**
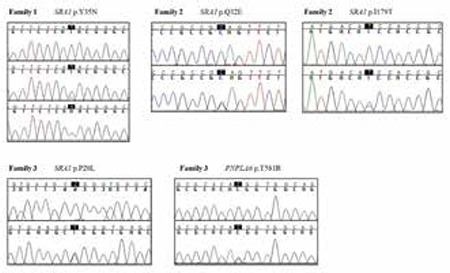
Results of automated DNA sequencing for SRA1 mutations in the three families. Top, middle, and bottom pictures show mutations in Family 1, 2, and 3, respectively. In Family 1 picture, top, middle, and bottom lines indicate wild type, heterozygous, and homozygous mutations, respectively. In Families 2 and 3 pictures, top and bottom lines indicate wild type and heterozygous mutations, respectively. In addition, the PNPLA6 mutation in Family 3 is also shown in the same order

**Family 1:** A genome-wide SNP analysis identified two regions of homozygosity common to the affected siblings but not found in the parents. Two autozygous regions spanned on chromosome 2 from 132159998 to 142605624 and on chromosome 5 from 107034345 to 161513613. Analysis of targeted exome sequencing data for the autozygous regions revealed that the only plausible candidate variant to account for the phenotype is in the SRA1 gene. A subsequent whole exome sequencing data on the proband were analyzed with particular attention to these autozygous regions by filtering for homozygous variants that are in the coding or splice regions and of minor allele frequency less than 1% or previously undescribed. Our analyses singled out a missense mutation in the SRA1 gene in the larger autozygous region on chromosome 5. We confirmed the presence of this nonsynonymous mutation in the coding sequence of SRA1 gene (HGNC: 11281) in the proband (Figure 2) by Sanger sequencing. The proband and his affected sibling were homozygous for a T-to-A change at cDNA nucleotide 103 (NM_ 001035235.3: c.T103A), leading to the substitution of tyrosine at residue 35 for asparagine (NP_001030312.2: p.Y35N). Their parents both were heterozygous for this mutation. Y35 is among phosphorylated residues. SIFT predicts that this substitution would affect the protein function with a score of 0.00 and PolyPhen-2 predicted this variant probably damaging. In addition, this variant was neither found in 100 ethnically matched healthy adult controls, 110 in-house whole exomes, nor in 1000 genomes, Exome Variant Server or in the ExAC databases. Besides, this variant was not seen in the Turkish whole exome database consisting of over 1000 Individuals’ data at TÜBİTAK-BİLGEM. Genotyping by Sanger screening and/or whole exome sequencing in search of additional SRA1 mutations in 136 IHH families revealed two other families with SRA1 mutations. In view of the co-occurrence of a PNPLA6 mutation in family 3, we thoroughly screened the affected individuals in family 1 and 2 for PNPLA6 mutations by Sanger sequencing and did not find any suspicious variants.

**Family 2:** A whole exome sequencing in the proband revealed two heterozygous variants: a C-to-G change at cDNA 94 predicting substitution of glutamine at residue 32 for glutamic acid, p.Q32E, rs35610885 and a T-to-C change at cDNA 536 predicting substitution of isoleucine at residue 179 for threonine, p.I179T, rs14810885 (Figure 2). Both of these variant were predicted to be harmful by SIFT and PolyPhen-2. In addition, these variants were not found in 100 ethnically matched healthy adult controls, 110 in-house exomes, or in 1000 genomes, or Exome Variant Server. The allele frequency rates of the p.Q32E and an in p.I179T variants are 0.007 and 0.0007 in ExAC; the allele frequency rates in Turkish whole exome database were 0.004 and 0.006, respectively.

**Family 3:** A Sanger screening for SRA1 showed that the proband and his affected brother had a heterozygous mutations (a C-to-T change at cDNA 59 predicting substitution of proline at residue 20 for leucine, p.P20L) (Figure 2). This variant was predicted to be harmful by both SIFT and PolyPhen-2. A whole exome sequencing in the proband revealed a heterozygous variant in PNPLA6 (HGNC: 16268, NM_006702; a c.C1742G leading to p.T581R) in both siblings (Figure 2) in addition to confirming the SRA1 variant described above. As can be seen in the pedigrees, the IHH phenotype segregated with co-occurrence of these two heterozygous mutations in the nuclear family, except for the eldest sibling who appears to be unaffected but carries the two variants. The whole exome sequencing in the proband showed no other mutations in known IHH genes. In addition, these variants were neither found in 100 ethnically matched healthy adult controls, 110 in-house exomes, 1000 genomes, or Exome Variant Server. The PNPLA6 variant was seen once in the ExAC while none in Turkish whole exome database respectively. The SRA1 variant is seen in 12 (allele frequency <1/1000-10.000) and once in the ExAC and Turkish whole exome database, respectively.

None of the probands from those three families harbored mutations in known IHH and Kallmann genes (5).

**Figure 3:**
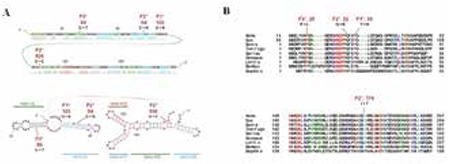
Position of the mutations in the ribonucleic acid and protein products. A. Positions of mutations in conserved SRA secondary structures. Conserved SRA secondary structures (Helices H1-2 and H15-18) identified by Novikova et al (28) and corresponding to the regions containing the observed mutations are depicted (28). Nucleotides are numbered using the first human “A” from the first AUG codon as 1. The exact location of the mutations observed in Family 1 (F1*, 103), Family 2 (F2*, 94 and 536), and Family 3 (F3*, 59) are indicated. Please note that all mutations but F1* affect nucleotides involved in conserved helices and might contribute to their stability. Shown are Dot bracket notation (top) and Plain secondary (bottom) structures. Graph generated by The Vienna ribonucleic acid (RNA) website (http://rna.tbi.univie.ac.at/cgi-bin/RNAfold.cgi). B. Position of mutations in conserved SRAP protein sequences. Two portions of the sequences of SRAP from Homo sapiens (NP_001030312.2, 236 aa), Susscrofa (XP_003124061.1, 280 aa), Daniorerio (NP_001002047.1, 210 aa), Takifugurubripes (XP_011609562.1, 264 aa), Gallus gallus (NP_001288615.1, 219 aa), Xenopuslaevi (NP_001107371.1, 227 aa), Lottiagigantea (XP_009055386.1, 279 aa), Bombyxmori (XP_004922978.1, 201 aa), and Daphnia pulex (EFX89230.1, 203 aa), which correspond to the region containing the mutations found in this study have been aligned. The numbers correspond to the positions of the side amino acids of the sequence shown. Amino acids identical, strongly similar, and weakly similar are colored in red, green, and blue, respectively. The top and bottom regions depicted correspond to the first and second phylogenetically conserved portion of SRAP, respectively (23). The exact location of the mutations observed in Family 1 (F1*, Y to N, 35), Family 2 (F2*, Q to E, 32 and I to T, 179), and Family 3 (F3*, P to L, 20) are indicated. Please note that these mutations modify amino acids that are identical in all chordata

Highly conserved positions of the mutations in the ribonucleic acid (RNA) and protein products of SRA1 are shown in Figure 3.

**Y35N Mutation Impairs SRA Mediated Estrogen Receptor Alpha (ESR1) Transactivation**

SRA1 gene products are known to potentiate ligand-dependent transcription of several nuclear receptor transcription factors as measured by response-element driven luciferase activity under standard luciferase reporter assay conditions (6,8,9,10,11). In particular, SRA has been shown to co-activate estradiol-induced estrogen receptor alpha (ESR1) transcription of luciferase reporters whose expression is under control of the estrogen-response-elements derived from the PS2 gene (PS2-ERE) (6,9,10). We have addressed whether the mutant found in family 1 (SRA-Y35N) could differentially co-regulated estrogen-dependent ER-alpha activity compared to wild-type SRA (SRA-WT). HeLa cells were co-transfected with an ERE-luciferase, ER-alpha, and either SRA-WT, SRA-Y35N, or SRA-MET7 control construct. This control corresponds to an artificial SRA sequence unable to encode functional SRA RNA due to extensive silent mutations at codon wobble positions, nor SRA protein (SRAP) as a result of site-directed conversion of all SRA methionine residues to leucines (6,12). Cell were subsequently treated with estradiol (+E2) or ethanol (cont) as described in the Material and Methods section.

**Figure 4:**
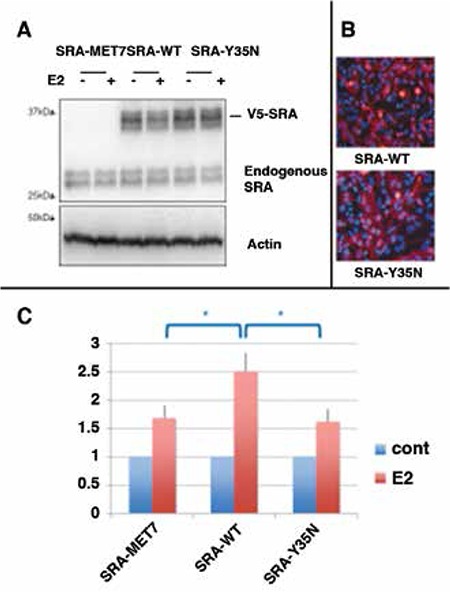
Functional analyses of SRA1 mutant p.Y35N A) Levels of SRAP expression protein extracts of transfected HeLa cells. Equal volumes of luciferase assay extracts from HeLa cells transfected with Estrogen Receptor-α (ESR1ESR1) and PS2-ERE luciferase reporter plasmids, and either control (SRA-MET7), wild-type (SRA-WT), or mutant (SRA-Y35N) SRA plasmids, were subjected to western blot analysis using anti-SRAP (743, Bethyl Laboratories) and anti-Actin antibodies (Abcam). Shown is a representative blot displaying equal levels of exogenous V5-epitope tagged SRAP (~35kDa) products in both SRA-WT and SRA-Y35N but not control SRA-MET7 transfected cell lysates. B) Pancellular localization of wild-type versus Y35N SRAP. HeLa cells were transiently transfected with either V5-epitope tagged SRA-WT or SRA-Y35N constructs and exogenous SRAP (Red) expression was observed by indirect immunofluorescent microscopy using anti-V5 (Life Technologies) primary antibody followed by anti-Mouse-Alexa555 (Life Technologies). Cells were counterstained with Dapi to visualize nuclei (Blue). C) SRA-Y35N mutation results in impaired estradiol induced ESR1 transactivation of PS2-ERE luciferase reporter. HeLa cells were co-transfected with Estrogen Receptor-α (ESR1), PS2-ERE luciferase reporter, and either control (SRA-MET7), wild-type (SRA-WT), or mutant (SRA-Y35N) SRA plasmids 24 h prior to being treated with estradiol (+E2) or ethanol (cont). Data were normalized as detailed in the Materials and Methods section. Error bars represent standard deviation for n=4. Unpaired 2 tailed student’s t-test was performed to test for significant difference among different conditions (*represents p<0.05)

We checked that SRA-WT and mutant SRA-Y35N levels reached in these experiments were similar. As shown in Figure 4A, Western blot analysis of protein extracts from cells transfected with either SRA-WT or SRA-Y35N showed that transfected cells express similar levels of endogenous SRAP and exogenous mutant proteins. We also checked that SRA-WT, an SRA-Y35N mutant protein, had similar localization (Figure 4B), i.e. cytoplasmic and nuclear, as previously described (13,14).

As shown in Figure 4, in the control SRA-MET7 and ESR1 co-transfected HeLa cells, estradiol treatment resulted in an approximate 1.6 fold increase in luciferase activity over that obtained from control transfected cells treated with ethanol vehicle alone (t-test, p<0.05) (Figure 4C, bars 1 vs 2). By comparison, co-transfection with SRA-WT resulted in a more pronounced and significant 2.5 fold increase in luciferase activity in the presence of estradiol (t-test, p<0.01) (compare bars 3 vs 4). These data are consistent with previously published results that indicate wild-type SRA to be an ESR1 co-activator (6). In contrast, SRA-Y35N did not increase the action of estradiol in this system, as indicated by similar luciferase activity in SRA-MET7 negative control and SRA-Y35N transfected cells (compare bars 2 and 6).

Overall, these data indicate that the mutant Y35N SRA is functionally different than the wild-type SRA and is unable to co-activate ER-alpha under these luciferase reporter assay conditions indicating a loss of function in this specific assay.

## DISCUSSION

In this article, we report three independent families in which affected siblings with inactivating mutations in SRA1 suffer from IHH. We identified and confirmed these mutations by a variety of genetic methods including candidate screening, genome-wide SNP genotyping and autozygosity mapping, targeted exome sequencing, and whole exome sequencing. Extreme rarity of these variants was confirmed not only in the international databases but also in in-house and national ones.

The products of this gene, SRA RNA and SRAP protein, define a very intriguing bi-faceted genetic system where both RNA and protein products of the same gene have the potential to play specific and sometimes overlapping roles in cell biology (15).

The steroid receptor RNA activator (SRA) was originally identified as a functional non-coding RNA involved in the regulation of gene expression by steroid receptors (8,12,16). It is now established that this RNA forms complexes, through critical secondary structures and loops, with a wide range of molecules including, but not limited to, multiple nuclear receptors, nuclear receptors co-regulators, proteins involved in gene silencing and gene insulation (8,11,17,18,19,20,21,22,23,24). Subsequently, some SRA transcripts were found to be able to encode for a protein, now referred to as the Steroid Receptor RNA Activator Protein (SRAP) (13,25,26). This made SRA1 the first gene able to encode for both a functional RNA and a protein (26). SRAP, as its RNA counterpart, has now been known to positively regulate the activity of steroid receptor such as the androgen receptor (AR) and the estrogen receptor (25,27).

We showed in this study that while WT-SRA acted, as expected, as an ER-alpha co-activator in a reporter assay, the mutation identified in Family 1 patients elicited a significantly decreased estradiol dependent ER-alpha activity. Notably, mutations in this study are located, in the protein sequence, within the two SRAP domains highly conserved among chordate (15,23). These mutations are also positioned in the Helix 1, 2, and 15 of the non-coding SRA sequence, which are highly conserved in all chordate (28). As outlined earlier, it has been shown that SRA conserved secondary structures were critical for its functional properties (12). Overall, the fact that the mutations identified here lay within both RNA and protein conserved regions emphasize the likelihood that they could have a profound effect on SRA and/or SRAP action, whatever the exact relevant mechanisms are involved.

IHH could be caused by defects in the hypothalamus and/or the pituitary. In both tissues, sex steroid receptors, ER-alfa and AR, are expressed. These receptors sense and accordingly respond to peripheral sex steroid levels in a negative feed-back pattern. Thus, inactivating mutations in SRA1, a known coactivator of ER-alfa (as once again shown in the control experiments in this study), should result in increased but not decreased gonadotropin levels. This would not be consistent with the phenotype of IHH seen in our patients. Indeed, in the rare cases of male and female patients who have inactivating ER-alfa mutations, there is a clear clinical picture of hypergonadotropic hypogonadism (29,30). Therefore, inactivating SRA1 mutations in our patients in this study must have caused hypogonadotropic hypogonadism by a mechanism other than via a decreased ER-alfa coactivation either at the hypothalamic or the pituitary level. When we stimulated the proband in Family 1 with GnRH in an extended fashion, the patient did not show a noticeable LH response, suggesting that the response of the pituitary gland to GnRH is compromised, thus we focused on the pituitary as the site of dysfunction leading to IHH (31).

Vertebrate members of the nuclear receptor NR5A subfamily, which includes steroidogenic factor 1 (SF-1) and liver receptor homolog 1 (LRH-1), regulate crucial aspects of development, endocrine homeostasis, and metabolism. In the pituitary, both LRH1 and SF-1 regulate the expression of the gonadotropins (32,33,34) and of the GnRH receptor (32,35). Mice with pituitary-specific disruption of SF-1 have markedly diminished levels of pituitary gonadotropins modeling hypogonadotropic hypogonadism (36). DAX-1 (NR0B1), a close partner of SF-1, acts as an adaptor that recruits other factors, such as the nuclear receptor corepressors to SF-1. In humans, inactivation mutations of DAX-1 are well known to cause hypogonadotropic hypogonadism (37). Notably, the endogenous SRA is required for the synergistic enhancement of SF-1 transcriptional activity by Dax-1. Taken together, it appears that SRA1 regulates SF-1 target gene expression by functioning as a coactivator in association with Dax-1 (11). Thus, reduced SRA1 activity due to inactivating mutations found in this study would result in diminished SF-1/LRH-1 effect leading to IHH, in parallel to the mechanism by which IHH is caused by inactivating DAX-1 mutations.

Interestingly, in one of the families (Family 3), we observed mutations in two genes, i.e. SRA1 and PNPLA6. Digenic inheritance is a well-established phenomenon of IHH, accounting for about 10% of all cases (38,39). With the increase in number of unbiased comprehensive genetic studies such as whole exome sequencing, it is now further appreciated that oligogenic inheritance is quite common in Mendelian disorders (40). Recently, a dedicated database for digenic inheritance has been established, in which IHH is listed along with other well-known oligogenic phenotypes such as non-syndromic hearing impairment (41).

In digenic inheritance, gene pairs are associated with one another by sharing pathway membership in about 20% of the time (41). We recently described patients with Gordon-Holmes syndrome (IHH and cerebellar ataxia) due to inactivating PNPLA6 mutations (42). PNPLA6 encodes for neuropathy target esterase (NTE), a lysophospholipase that maintains intracellular phospholipid homeostasis by converting lysophosphatidylcholine to glycerophosphocholine (42). We also demonstrated that inhibition of NTE activity in the LβT2 gonadotrope cell line, which represents the pituitary gonadotropes, diminished LH response to GnRH by impaired LH release from pituitary gonadotropes leading to IHH. Thus, the sites of action of SRA1 and PNPLA6 seem to overlap at the pituitary gonadotropes, which suggests that dysfunction of these two gene variants potentialize or are additive to each other. Even more interestingly, there is ample evidence in the literature to show that phospholipids including certain species of lysophosphatidylcholine are ligands for SF-1/LRH-1, potentially placing two genes (i.e. SRA1 and PNPLA6) in the same pathway (43,44). In fact, phospholipids may represent a potential link between metabolism and reproductive function (45). Although a pituitary site of action seems probable, a hypothalamic involvement cannot be ruled out as there are publications supporting this contention. Most notably, both LRH-1 and DAX-1 (46) are expressed in the arcuate nucleus and LRH-1 provides a stimulus for kisspeptin activation in the GnRH pulse generator (47).

As for the unusual clinical and laboratory features of these families, the proband in Family 2 and the younger brother (II-4) in Family 3 had undescended testicles and micropenis in infancy suggesting a profound prenatal undervirilization due to a severe IHH. But later, at a delayed age, they went through puberty with or without intervention. Spontaneous or induced regain of central gonadal function or reversibility is seen in about 10 percent of all IHH cases (48), even in the most severe cases of congenital IHH (49). In Family 3, the older sister (II-1) appears to be unaffected despite carrying the same two variants as her affected brothers. Like her younger brother, she may have gone through spontaneous recovery. It should be noted however, that similar variability of phenotypes from IHH to delayed puberty to even normal timing in persons carrying the same genotype within the same family has been repeatedly observed (50,51,52,53). Alternatively, a third mutated gene or a copy number variation among others could have provided an explanation for the genotype/phenotype discrepancy in this family, but we have not been able to find one.

Lastly, a global knock-out of SRA in the mouse has been recently reported. This model mouse protects against diet-induced obesity and improves whole body glucose homeostasis probably via its action as a PPARγ coactivator. The SRA−/− mice appeared “normal” with no specific information regarding their reproductive function provided (54).

In conclusion, it is evident from the studies reported here that inactivating mutations of the SRA1 gene cause complete normosmic IHH, hence pubertal failure in humans, and we would argue that proper function of SRA1 is a critical element of the central gonadal function in humans. It is tempting to speculate that SRA1, an intriguing gene whose products functioning both as a protein and a noncoding RNA, may in part account for the complexity, versatility, and elusiveness of the pubertal process, especially when one considers the fact that actions of nuclear receptor coregulators can spatially and temporally vary to become activators or repressors of the target nuclear receptors depending on the cellular and promoter context.

## ACKNOWLEDGMENT

This work was supported by the Scientific and Technological Research Council of Turkey (TÜBİTAK) (Project no: 113S962) and by the Çukurova University Scientific Research Projects. The Laboratory of EL is currently funded by the Canadian Breast Cancer Foundation.

We thank the Advanced Genomics and Bioinformatics Research Center (IGBAM) for checking the variant frequency in their in-house Turkish whole exome database at the TÜBİTAK-BİLGEM.

The authors thank Dr. Sergio R. Ojeda, Dr. Alejandro Lomniczi, and Dr. Juan M. Castellano of OHSU Oregon National Primate Center for valuable discussions.

**Ethics**

Ethics Committee Approval: The Ethics Committee of the Çukurova University Faculty of Medicine approved, Informed Consent: Obtained from each participant or from the parents.

Peer-review: Internal peer-reviewed.

## AUTHORSHIP CONTRIBUTIONS

Concept: Leman Damla Kotan, A. Kemal Topaloğlu, Etienne Leygue, Design: Leman Damla Kotan, A. Kemal Topaloğlu, Etienne Leygue, Data Collection and/or Processing: Charlton Cooper, Şükran Darcan, Samim Özen, Yi Yan, Fatih Gürbüz, Eda Mengen, İhsan Turan, Ayça Ulubay, Gamze Akkuş, Bilgin Yüksel, Analysis and/or Interpretation: Ian M. Carr, Charlton Cooper, Mohammad K. Hamedani, Literature Research: Leman Damla Kotan, Fatih Gürbüz, Eda Mengen, İhsan Turan, Writing: Leman Damla Kotan, A. Kemal Topaloğlu, Etienne Leygue.

Financial Disclosure: The authors declared that this study received no financial support.

## Figures and Tables

**Table 1:**
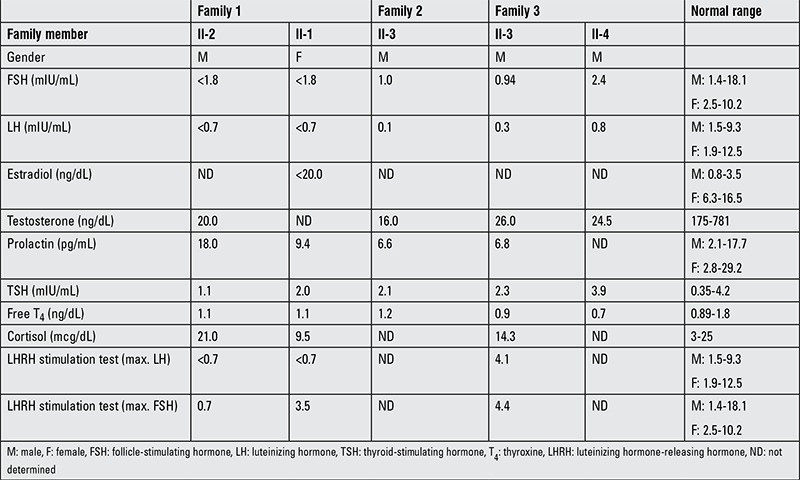
The clinical and hormonal features of the affected individuals in the three families
